# Buttiauxella cochleicola sp. nov., a novel coliform bacterium with β-d-glucuronidase activity, isolated from gastropods and drinking water

**DOI:** 10.1099/ijsem.0.007265

**Published:** 2026-08-13

**Authors:** Michael Hügler, Carolin Leister, Vera Aschmann, Monika Bösl, Lydia Grabner, Luisa Kurcz, Sarah Kirstein, Jacqueline Wolf, Richard L. Hahnke, Vera Thiel

**Affiliations:** 1 TZW: DVGW-Technologiezentrum Wasser, Karlsruher Str. 84, D-76139 Karlsruhe, Germany; 2 Leibniz-Institute DSMZ – German Collection of Microorganisms and Cell Cultures GmbH, Inhoffenstraße 7B, D-38124 Braunschweig, Germany

**Keywords:** *Buttiauxella*, coliform bacteria, drinking water, *Enterobacterales*, gastropoda, *β*-d-glucuronidase

## Abstract

Four strains of coliform bacteria were isolated from drinking water (strains A2-C1_F and A2-C2_NF) and gastropod samples (strains W03-F01^T^ and S04-F03). 16S rRNA gene sequence analysis showed the highest similarities to members of the genus *Buttiauxella* (>98%), indicating that all strains are members of this genus. Phylogenomic analyses revealed that strains W03-F01^T^, S04-F03 and A2-C1_F can be assigned to a putative new *Buttiauxella* species, while strain A2-C2_NF is a member of the species *Buttiauxella ferragutiae*. The strains W03-F01^T^, S04-F03 and A2-C1_F show fluorescence in the Colilert system, indicating unusual *β*-d-glucuronidase activity, typically considered indicative for *Escherichia coli* (and thus faecal contamination) in water quality analysis. The draft genome of strain W03-F01^T^ has a size of 4.79 Mb and a G+C content of 50.0 mol%. With average nucleotide identity and digital DNA–DNA hybridization values of 96.2 and 68.8%, respectively, strain W03-F01^T^ was most closely related to *Buttiauxella massiliensis* DSM 110695^T^. Additional extensive physiological, phenotypic and chemotaxonomic characterization of the strains and all reference strains confirmed the proposal of a novel species. Therefore, the strains W03-F01^T^, S04-F03 and A2-C1_F represent a novel species for which the name *Buttiauxella cochleicola* sp. nov. is proposed. The type strain is W03-F01^T^ (=DSM 113920^T^=CIP 112567^T^).

## Data Availability

The whole genome sequences of the new strains have been deposited with the NCBI database under the following accession numbers: strain W03-F01^T^, JAJSOT000000000.1; strain S04-F03, JAJSOS000000000.1; strain A2-C1_F, JAJSOU000000000.1; strain A2-C2_NF, JAJSOV000000000.1.

## Introduction

Worldwide, the hygienic-microbiological quality of drinking water is examined by regularly monitoring the presence of faecal indicator bacteria. Coliform bacteria and *Escherichia coli* are members of the *Enterobacteriaceae* (*Pseudomonadota*, *Gammaproteobacteria, Enterobacterales*) and common indicator bacteria in the examination of microbial drinking water quality. For their detection, *β*-d-galactosidase and *β*-d-glucuronidase activities are tested in membrane filtration-based water quality tests or most probable number methods like the Colilert system [1, 2]. The presence of *β*-d-glucuronidase activity is considered indicative for *E. coli* [3, 4], while non-*E. coli* coliform bacteria exhibit *β*-d-galactosidase activity only [5, 6]. *E. coli* is the most important indicator for faecal water contamination, and its presence is used as a proxy for contaminations with faecal waterborne pathogens, as *E. coli* is an important inhabitant of the gastrointestinal tract of humans and warm-blooded animals and not found in non-living environmental samples. In response to the detection of *E. coli* in drinking water, health authorities usually announce ‘boil-water advisories’ for the affected consumers [6]. In contrast, coliform bacteria are usually considered a general indicator for water quality, as although they are present in the faeces of different animals, they also occur or even proliferate in the environment, e.g. surface water bodies, soil, plants or invertebrates [7–9]. Coliform bacteria are often of environmental origin, and their presence in drinking water does not necessarily indicate faecal contamination through warm-blooded animals. Thus, in drinking water analysis, it is of great importance to differentiate between *E. coli* and other coliform bacteria.

The genus *Buttiauxella* belongs to the group of coliform bacteria, and strains of the genus have mainly been isolated from environmental sources, such as fresh water, soils and especially the intestines of snails and slugs [10]. They are considered as environmental bacteria not indicative of faecal contamination and generally possess *β*-d-galactosidase activity, while *β*-d-glucuronidase activity is missing. The genus *Buttiauxella*, first described by Ferragut *et al.* [11], comprises nine species with validly published names: *Buttiauxella agrestis* Ferragut *et al*. 1982 [11], *Buttiauxella brennerae* Müller *et al*. 1996 [12], *Buttiauxella ferragutiae* Müller *et al*. 1996 [12], *Buttiauxella gaviniae* Müller *et al*. 1996 [12], *Buttiauxella izardii* Müller *et al*. 1996 [12], *Buttiauxella noackiae* Müller *et al*. 1996 [12], *Buttiauxella selenatireducens* Zhang *et al.* 2024 [13], *Buttiauxella warmboldiae* Müller *et al.* 1996 [12] and *Buttiauxella massiliensis* Zgheib *et al.* 2022 [14]. Another described species is ‘*Buttiauxella chrysanthemi*’ Furlan *et al.* 2018, isolated from a soil sample of a chrysanthemum plantation [15]. Thus, *Buttiauxella* are facultatively anaerobic, chemoorganotrophic and psychrotolerant environmental bacteria that have been isolated from freshwater, soil and invertebrates. Especially the intestine of snails and slugs is considered an ecological niche for these bacteria [10, 12]. Human infections with *Buttiauxella* strains have rarely been reported [16, 17]. *B. massiliensis* DSM 110695^T^ is the only type strain that has been isolated from a clinical sample, namely from a human bone infection of an open fibula fracture, yet its pathogenic relevance remains unclear [14].

In this study, we report the isolation of four bacterial strains of the genus *Buttiauxella* from faeces samples of gastropods (strains W03-F01^T^=DSM 113920^T^=CIP 112567^T^ and S04-F03=DSM 113932=CIP 112568) and a drinking water sample (strains A2-C1_F=DSM 113933=CIP 112569 and A2-C2_NF=DSM 113919=CIP 112570). Three of the strains (W03-F01^T^, S04-F03 and A2-C1_F) were unusual, as they exhibited *β*-d-glucuronidase activity, commonly considered as indicative for *E. coli*. Our analysis showed that these strains represent a novel *Buttiauxella* species, herein proposed as *Buttiauxella cochleicola* sp. nov., with W03-F01^T^ (=DSM 113920^T^=CIP 112567^T^) as type strain.

## Methods

### Isolation

The strains W03-F01^T^ and S04-F03 were obtained from faeces samples of the gastropods *Helix pomatia* and *Arion vulgaris*, respectively, collected at Kraichtal-Gochsheim (49.104 N 8.744 E) (see [6] for further details). The strains A2-C1_F and A2-C2_NF were obtained from a water sample of a drinking water reservoir of a small village near Bruchsal, Germany (49.084 N 8.632 E), using Colilert-18/Quanti-Tray (IDEXX Laboratories) according to DIN EN ISO 9308-2 [1]. The strains were isolated from a fluorescent and yellow-coloured well (indicating *E. coli*) and a non-fluorescent yellow-coloured well (indicating coliform bacteria), respectively [5]. To obtain single colonies, liquid of the respective wells was transferred onto heterotrophic plate count (HPC) agar plates (Merck), recommended by German regulations (Deutsches Einheitsverfahren) and incubated for 24 h at 36 °C. Purification was achieved by continuous transfers on agar plates. The isolates were maintained on HPC agar plates (Merck) at 36 °C. The strains were also plated on chromocult coliform agar (Merck) to verify *β*-d-galactosidase and *β*-d-glucuronidase activity. Bacteria with *β*-d-galactosidase appear as salmon-dyed colonies, whereas bacteria with *β*-d-glucuronidase have a blue colour.

### Identification and phylogenetic analysis

Identification of the strains was done with MALDI-TOF MS on a MALDI Biotyper Microflex LT mass spectrometer using the extended direct transfer and the extraction method according to the manufacturer’s instructions (Bruker Daltonics GmbH) [8]. 16S rRNA genes were PCR-amplified using the primer pair 27F and 1492R [18]. All sequences were edited with ChromasPro Version 2.1.10. Phylogenetic analysis of 16S rRNA gene sequences and *β*-d-glucuronidase (UidA) protein sequences was conducted in mega X [19]. Alignment was performed using the multiple sequence alignment method Clustal W [20] and the parameters recommended by Hall [21]. Phylogenetic analysis was done using the maximum-likelihood method (Tamura–Nei model) with 1,000 replicates.

### Genome sequencing and phylogenomic analysis

The genomes of the four strains were sequenced and annotated as described in Leister and Hügler [5]. Genomic DNA of pure cultures was extracted using a FastDNA spin kit for soil (MP Biomedicals). Preparation of sequencing libraries was performed using a DNA prep kit (Illumina). The draft genomes were sequenced by 150 bp paired-end sequencing on an Illumina NextSeq 1000 instrument using NovaGene (Illumina). Reads were trimmed using Cutadapt v1.16.6 [22] and quality-controlled using FastQC v0.72. High-quality sequence reads were assembled *de novo* using Unicycler v0.4.6.0 [23]. Genome quality was assessed using CheckM [24]. Annotation was carried out using RASTtk v2.0 [25, 26] and the NCBI Prokaryotic Genome Annotation Pipeline v5.0 [27, 28].

Similar genomes were searched using the Similar Genome Finder of BV-BRC, available under https://www.bv-brc.org, using the Mash/MinHash algorithm [29, 30]. Genome-based phylogeny was conducted with the codon-tree pipeline in BV-BRC [29]. A tree from 1,000 genes was generated using single-copy cross-genera protein families analysing aligned proteins and coding DNA from single-copy genes with RAxML [31–33]. The genome sequence data were further uploaded to the Type (Strain) Genome Server, available under https://tygs.dsmz.de, for a whole genome-based taxonomic analysis, including digital DNA–DNA hybridization (dDDH) [34, 35]. Average nucleotide identity (ANI) was calculated with the OrthoANIu algorithm using the web service ANI Calculator from EzBioCloud [36, 37]. The genomes were checked for potential plasmids using PlasmidFinder [38]. Comparative genomics was done using the web-based tool PanExplorer [39].

### Morphology, physiology and chemotaxonomy characterization

Physiological and chemotaxonomic characterization was performed with strains W03-F01^T^, S04-F03, A2-C1_F, A2-C2_NF and the reference strains *B. agrestis* DSM 4586^T^, *B. brennerae* DSM 9396^T^, *B. ferragutiae* DSM 9390^T^, *B. gaviniae* DSM 9393^T^, *B. izardii* DSM 9397^T^, *B. noackiae* DSM 9401^T^, *B. warmboldiae* DSM 9404^T^ and *B. massiliensis* DSM 110695^T^. *B. selenatireducens* R73^T^ was included in phylogenetic analyses, yet physiological and chemotaxonomic data were taken from literature [13]. Culture-based and morphological characterization was carried out with cultures grown on HPC agar plates (Merck) or in tryptic soy broth (TSB, Merck). The Gram behaviour was tested with Bactident^®^ aminopeptidase test strips (Merck) and the reaction of cells towards a 3% potassium hydroxide solution. Oxidase activity was tested with oxidase test strips (Millipore), and catalase activity was tested by gas formation after saturation with hydrogen peroxide (Roth) on fresh biomass grown for 48 h on HPC agar. Biochemical properties, carbon source utilization and chemical sensitivity were determined using the Biolog GenIII MicroPlate according to the manufacturer’s instructions.

Growth at different temperatures, pH values and NaCl concentrations was tested for the strains W03-F01^T^, S04-F03, A2-C1_F and A2-C2_NF after incubation for 24 h, 48 h and 72 h on HPC agar and in liquid culture (LB bouillon, Merck). The tested temperatures were 2 °C, 4 °C, 12 °C, 22 °C, 25 °C, 30 °C, 36 °C, 42 °C, 45 °C and 48 °C. The pH range was tested between 4.5 and 11.5, using acetate (pH 4.5 and 5.5), phosphate (pH 6.5, 7.5, 8.5) and glycine buffer (pH 9.5, 10.5, 11.5). NaCl tolerance was investigated with different NaCl concentrations between 0.5 and 10% (w/v). Growth under anaerobic conditions was tested using AnaeroJar containers and gas packs (Oxoid).

Chemotaxonomic analysis (fatty acids, respiratory quinones and polar lipids) was performed with the four new strains and the above-mentioned reference strains. We determined the cellular fatty acid profiles with cells cultivated on TSB agar at 37 °C. The fatty acids were analysed after conversion to fatty acid methyl esters (FAMEs). Saponification, methylation and extraction were performed according to the protocol described by Sasser [40]. The FAME mixtures were separated by GC and quantified using a flame ionization detector. In subsequent analyses, FAMEs were identified based on retention time and mass spectra by GC-MS on an Agilent GC-MS 7000D system [41]. FAMEs were derivatized to dimethyl disulphide adducts to resolve the position of the single double bonds [42]. Furthermore, cyclo-positions were determined by the derivatization of their 4,4-dimethyloxazoline derivatives [43]. Respiratory quinones were extracted from cells grown on TSB medium and analysed via HPLC coupled to a diode array detector as described previously [41]. Polar lipids were extracted from freeze-dried biomass with a mixture of chloroform:methanol:0.3% NaCl and recovered into the chloroform phase (modified after [44]). Polar lipids were analysed via two-dimensional thin-layer chromatography, and total lipids were visualized by spraying plates with molybdophosphoric acid. Specific functional groups were further visualized with different spray reagents designed for defined functional groups [45].

## Results and discussion

### Identification and phylogenetic analysis

**Fig. 1.**
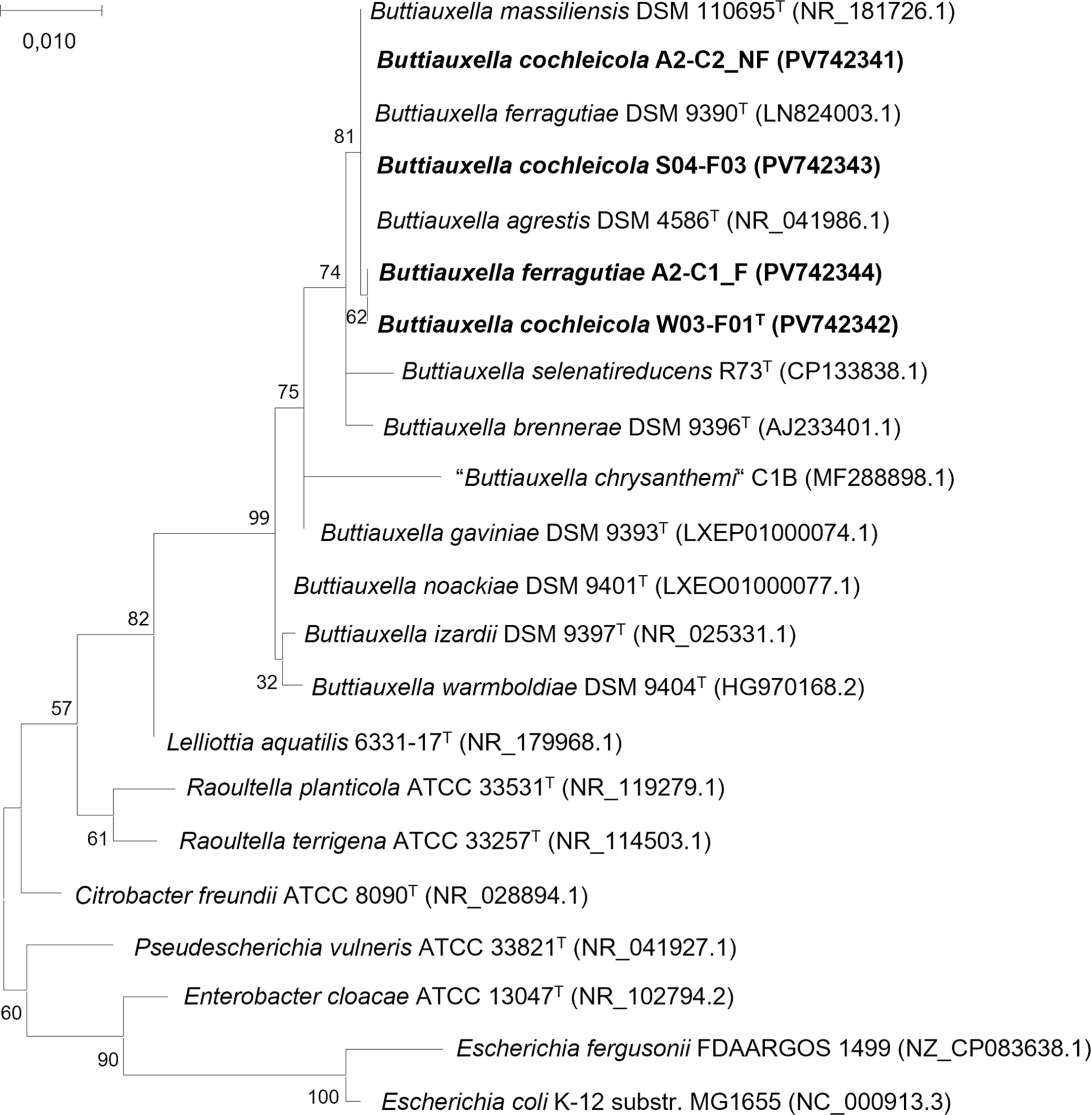
Phylogenetic tree based on based on 16S rRNA gene sequences inferred using the maximum-likelihood method and Tamura–Nei model [53]. Bootstrap values based on1,000 replications are shown. Scale bar: 0.01 substitutions per nucleotide.

Identification of the new strains using MALDI-TOF MS revealed *B. gaviniae* as the best match for strains W03-F01^T^, S04-F03 and A2-C1_F (score values 2.1 to 2.2), and *B. ferragutiae* as the best match for strain A2-C2_NF (score value around 2.3). The 16S rRNA gene exhibited the highest similarities (100%) with *B. agrestis* DSM 4586^T^, *B. ferragutiae* DSM 9390^T^ and *B. massiliensis* DSM 110695^T^, yet sequence similarities above 98% were detected among all described members of the genus *Buttiauxella* (Fig. 1). Thus, based on 16S rRNA gene sequence alone, species differentiation is not possible within the genus *Buttiauxella*, as for the *Enterobacteriaceae* in general [46].

### Genomic properties and phylogenomic analysis

The genomic properties of the new strains and reference strains are shown in Table 1. Genome quality assessment revealed highly complete draft genomes without relevant contaminations (Table S1, available in the online Supplementary Material). The draft genomes of strains W03-F01^T^, S04-F03 and A2-C1_F were composed of 25, 36 and 36 contigs with total sizes of 4.79, 4.80 and 4.83 Mbp, respectively, which is within the range of the reference genomes of this genus. The G+C contents were 50.0 mol%, 50.1 mol% and 50.1 mol%, and genome annotation resulted in 4,350, 4,365 and 4,406 protein CDSs, respectively. Strain A2-C2_NF had a larger genome of 5.22 Mbp with a slightly higher G+C content (50.6 mol%) and a higher number of CDSs (4,747) (Table 1) [5].

**Table 1.** Characteristics and accession numbers of genomes

Bacterial species	Strain	Sample	*uidA*	Genome coverage (x)	Genome size (Mb)	No. of contigs	*N_50_* (kb)	G+C content (mol%)	No. of CDSs	WGS accession no.
*B. cochleicola*	W03-F01^T^	*H. pomatia*	+	34.1	4.79	25	398.1	50.0	4,350	JAJSOT000000000
*B. cochleicola*	S04-F03	*A. vulgaris*	+	36.3	4.80	36	310.9	50.1	4,365	JAJSOS000000000
*B. cochleicola*	A2-C1_F	Water	+	30.9	4.83	36	290.5	50.1	4,406	JAJSOU000000000
*B. ferragutiae*	A2-C2_NF	Water	−	33.7	5.22	61	324.5	50.6	4,747	JAJSOV000000000
*B. massiliensis*	DSM 110695^T^	Clinical	−	−	4.88	50	353.0	49.6	4,560	CABVOV000000000
*B. gaviniae*	DSM 9393^T^	Snail	−	37	5.1	133	223.6	49.5	4,687	LXEP00000000
*B. noackiae*	DSM 9401^T^	Snail	−	35	4.8	104	143.5	49.5	4,391	LXEO00000000
*B. brennerae*	DSM 9369^T^	Snail	−	38	4.8	88	278.5	50.5	4,347	LXER00000000
*B. ferragutiae*	DSM 9390^T^	Soil	−	177	5.3	1	5,300	50.5	4,665	CP093332.1
*B. selenatireducens*	R73^T^	Soil	−	350	5.2	1	5,200	50.5	4,611	CP133838.1
*B. warmboldiae*	DSM 9404^T^	Snail	−	98	4.3	135	55.7	52.5	3,870	RPOH00000000
*B. agrestis*	DSM 4586^T^	Soil	−	15	4.7	93	135.6	51.0	4,287	JMPI00000000
*B. izardii*	DSM 9397^T^	Snail	−	280	4.8	91	91.3	51.3	4,319	QZWH00000000

Search for similar genomes using the Similar Genome Finder Tool revealed *B. massiliensis* DSM 110695^T^ as the most closely related genome to strains W03-F01^T^, S04-F03 and A2-C1_F, followed by the genomes of *B. gaviniae* and *B. noackiae* strains. Other genomes available in public databases were less related and did not yield any further phylogenomic insights. Consequently, only the results of type strains are shown.

**Fig. 2.**
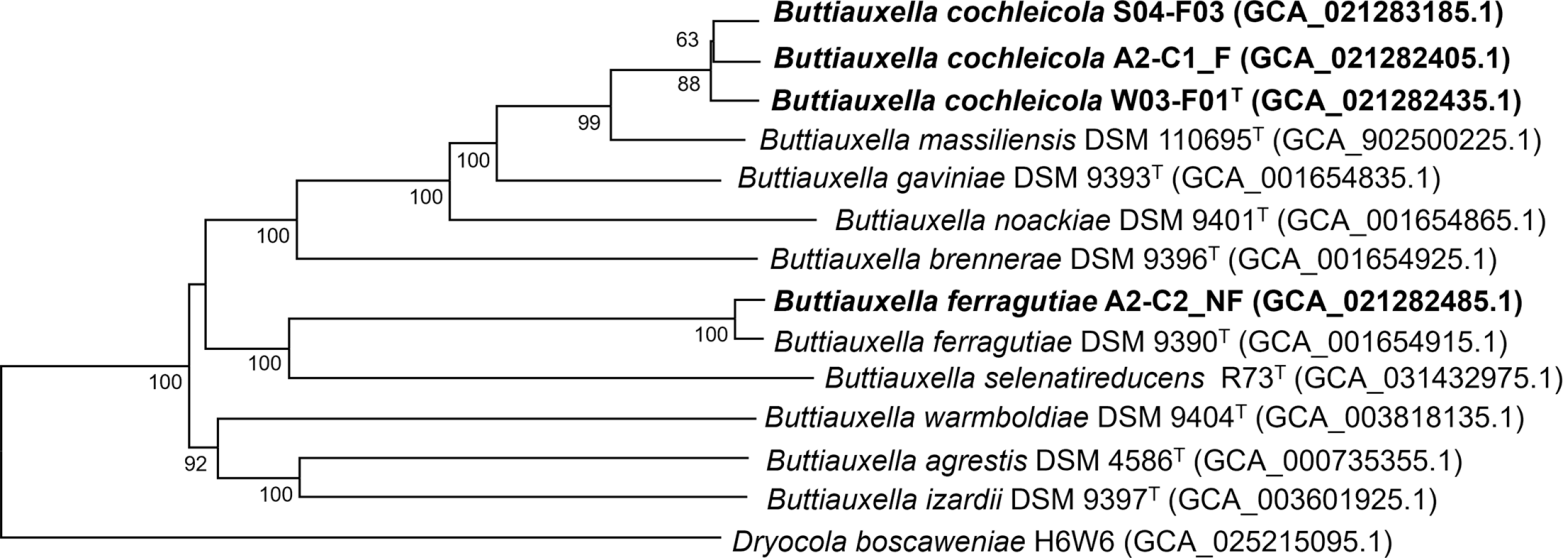
Phylogenetic tree inferred with FastME 2.1.6.1 [54] from GBDP distances calculated from genome sequences. The branch lengths are scaled in terms of GBDP distance formula *d*_*5*_. The numbers above the branches are GBDP pseudo-bootstrap values >60% from 100 replications, with an average branch support of 92.3%. The tree was rooted with *Dryocola*.

The results of genome-based taxonomic analysis are shown in Table 2 (ANI and dDDH); Figs 2 (Genome blast Distance Phylogeny), S1 (genome-based phylogeny), S2 (pan-genome-based phylogeny) and S3 (pan-genome visualization); and Table S2 (gene presence/absence).

**Table 2.** Comparative ANI and dDDH between *Buttiauxella* species Strains: W03-F01^T^ (1), S04-F03 (2), A2-C1_F (3), A2-C2_NF (4), *B. agrestis* DSM 4586^T^ (5), *B. brennerae* DSM 9396^T^ (6), *B. ferragutiae* DSM 9390^T^ (7), *B. gaviniae* DSM 9393^T^ (8), *B. izardii* DSM 9397^T^ (9), *B. massiliensis* DSM 110695^T^ (10), *B. noackiae* DSM 9401^T^ (11), *B. warmboldiae* DSM 9404^T^ (12) and *B. selenatireducens* R73^T^ (13).

		ANI (%)												
		1	2	3	4	5	6	7	8	9	10	11	12	13
dDDH	1		**98.69**	**98.69**	85.23	85.88	87.84	85.28	93.45	86.11	**96.22**	91.39	85.20	84.66
(%)	2	**88.7**		**98.72**	85.26	86.09	87.71	85.42	93.38	86.06	**96.17**	91.34	85.06	84.48
	3	**88.8**	**89.5**		85.31	85.91	87.58	85.53	93.40	86.12	**96.08**	91.51	85.26	84.74
	4	30.3	30.2	30.3		85.60	85.91	**99.20**	85.39	85.21	85.51	85.11	84.84	86.78
	5	30.9	30.9	31.0	30.2		85.93	85.63	85.82	88.59	86.18	85.48	86.27	84.51
	6	34.4	34.5	34.5	30.9	31.1		85.78	89.48	85.31	87.83	86.83	84.90	84.76
	7	30.2	30.2	30.3	**93.5**	30.3	30.8		85.40	85.25	85.42	85.17	84.66	86.71
	8	53.1	53.1	53.0	30.2	30.6	39.1	30.2		85.55	93.90	91.26	85.08	84.67
	9	31.2	31.1	31.2	29.8	35.8	30.1	29.8	29.8		86.54	84.96	86.04	84.46
	10	**68.8**	**68.8**	**68.4**	30.1	31.3	34.8	30.2	54.7	32.0		91.29	85.21	84.64
	11	45.1	44.9	44.9	29.8	29.8	32.6	29.8	44.5	29.4	44.7		84.89	84.47
	12	29.6	29.7	29.7	29.0	31.3	29.3	28.9	29.4	31.3	29.7	29.5		84.12
	13	28.9	28.9	28.9	33.0	28.9	29.0	33.1	28.9	28.9	29.0	28.9	28.4	

The ANI and dDDH values among the three strains W03-F01^T^, S04-F03 and A2-C1_F were ≥98.69% and ≥88.7%, respectively, indicating them to belong to the same species. In comparison to the type strain genomes of described *Buttiauxella* species, ANI and dDDH values of strains W03-F01^T^, S04-F03 and A2-C1_F were well below 95–96 and 70%, respectively, the generally accepted values for species boundary [47], except for *B. massiliensis* DSM 110695^T^. Comparative ANI between strains W03-F01^T^, S04-F03 and A2-C1_F and *B. massiliensis* DSM 110695^T^ revealed values between 96.08 and 96.22%, which are slightly above the threshold for species separation [48]. In contrast, dDDH computations yielded values below the commonly accepted threshold of 70% for species demarcation (68.8, 68.8 and 68.4%, respectively) (Table 2).

Further genome-based analyses, including GBDP (Fig. 2), genome-based phylogeny calculating a tree from 1,000 genes (Fig. S1) and pan-genome phylogeny (Fig. S2), support the dDDH results. Genome-, gene- and pan-genome-based phylogenies show a clear separation between strains W03-F01^T^, S04-F03 and A2-C1_F and *B. massiliensis* DSM 110695^T^. Gene presence/absence analyses (Fig. S3 and Table S2) provide further complementary evidence, indicating 325 genes present in the 3 novel isolates, but absent in *B. massiliensis* DSM 110695^T^, and 553 genes found in *B. massiliensis* DSM 110695^T^ but not in the 3 new isolates. Thus, based on the polyphasic analyses, strains W03-F01^T^, S04-F03 and A2-C1_F represent a new species of the genus *Buttiauxella* clearly distinct from *B. massiliensis* DSM 110695^T^ (see also taxonomic conclusion below).

As no genome sequence of ‘*B. chrysanthemi*’ C1B is available in the public databases, phylogenetic analyses of 16S rRNA (Fig. 1) and *rpo*B genes were used to compare strains W03-F01^T^, S04-F03 and A2-C1_F to ‘*B. chrysanthemi*’ C1B. The genes showed similarities of 98.03% (16S rRNA) and 95.53% (*rpo*B), respectively. These values are clearly below the proposed cut-off of less than 98.5% (16S rRNA) and 97.7% (*rpo*B), indicating that W03-F01^T^, S04-F03 and A2-C1_F cannot be assigned to the species ‘*B. chrysanthemi*’ C1B [49, 50].

For strain A2-C2_NF, the ANI and dDDH analyses clearly assign this strain to the species *B. ferragutiae* (values of 99.20% (ANI) and 93.5% (dDDH), respectively (Table 2)), confirming the results of MALDI-TOF MS identification.

### Presence of indicative enzymes *β*-d-galactosidase and *β*-d-glucuronidase

Coliform bacteria produce the enzyme *β*-d-galactosidase as part of the lactose fermentation pathway. Thus, it is not surprising that the genome sequences of the new strains confirmed the presence of the genes coding for *β*-d-galactosidase (*lacZ*) in all four strains (accession numbers: MCE0799391.1, MCE0812344.1, MCE0845361.1 and MCE0826947.1, respectively). In addition, the *uidA* gene coding for *β*-d-glucuronidase was present in the strains W03-F01^T^, S04-F03 and A2-C1_F (MCE0800444.1, MCE0812571.1 and MCE0846528.1, respectively), confirming biochemical results (fluorescence in the Colilert-18/Quanti-Tray test). It was not found in the genome of strain A2-C2_NF, correlating with the observed lack of fluorescence in the Colilert-18/Quanti-Tray assay. Thus, genome sequences confirmed the results of the Colilert-18/Quanti-Tray tests.

**Fig. 3.**
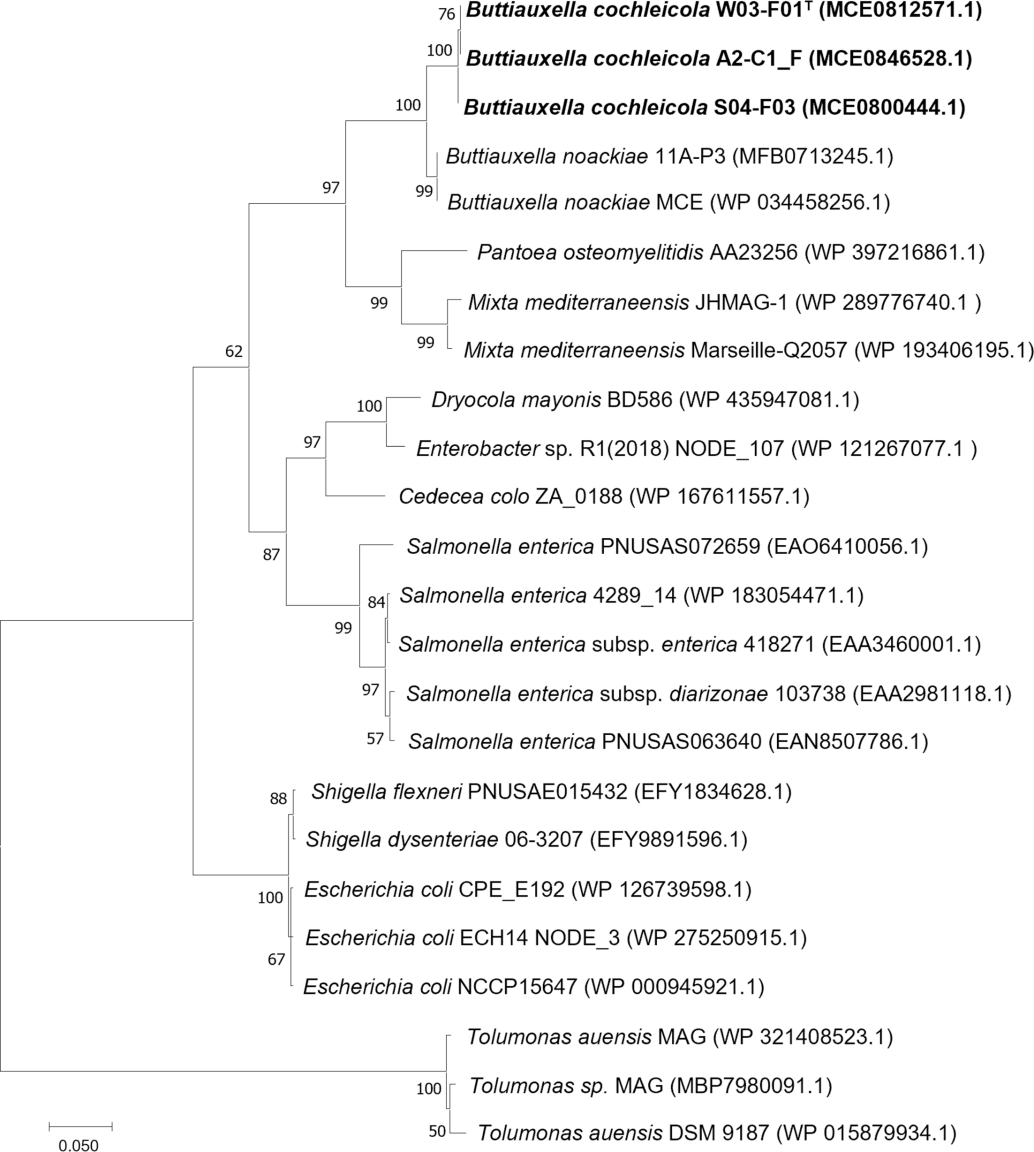
Phylogenetic tree based on *β*-d-glucuronidase protein sequences inferred using the maximum-likelihood method and Tamura-Nei model [53] with bootstrap values based on 1,000 replications. Scale bar of 0.05 substitutions per amino acid position.

The *uidA* gene has further been detected in two additional *Buttiauxella* spp. isolates/genomes, *B. noackiae* MCE [3] and *B. noackiae* 11 A-P3. Fig. 3 shows the phylogenetic analysis of the *β*-d-glucuronidase protein sequences. Fig. S4 gives the genomic context of the *uid*-genes in the respective *Buttiauxella* strains, *E. coli*, *Shigella* and *Salmonella*. *β*-d-Glucuronidase of strains W03-F01^T^, S04-F03 and A2-C1_F clusters together with *β*-d-glucuronidase of the two other *Buttiauxella* strains and clearly differs from the enzyme sequences of *E. coli* and *Salmonella* (Fig. 3) [6]. As in *E. coli*, the *uidA* gene is organized in an operon (*uidABC* with the transcriptional repressor *uidR* upstream) (Fig. S4). The operon is most likely located on the chromosome, as no hints for plasmids could be found. Furthermore, potential transposases could not be detected in the vicinity of the operon.

### Phenotypic, physiological and chemotaxonomic characterization

The results of the phenotypic, physiological and chemotaxonomic characterization of the different strains and *Buttiauxella* reference strains are shown in Tables 3, 4, 5 and S3, respectively. All four isolates were Gram-negative; oxidase activity was negative, and catalase activity was positive. They grew aerobically and facultatively anaerobically on HPC agar. In aerobic atmosphere, they formed beige, smooth colonies with a diameter of 2–4 mm after 48 h at 36 °C, thus showing a typical colony morphology of *Enterobacteriaceae*. Plating on chromogenic coliform agar plates revealed different colony colours for the strains. Strains W03-F01^T^, S04-F03 and A2-C1_F, isolated from fluorescent wells, appeared light blue, indicative for *β*-d-glucuronidase activity, whereas strain A2-C2_NF, isolated from a non-fluorescent well, appeared salmon coloured, thus typical for coliform bacteria with *β*-d-galactosidase activity [6]. The rod-shaped cells were ~1 µm wide and 2–4 µm long. The optimal growth temperature for strains W03-F01^T^, S04-F03 and A2-C1_F was 30 °C. The strains showed good growth between 12 and 36 °C and grew weakly at 4 and 42 °C. All isolates grew between pH 5.5 and 10.5 with optimal growth around pH 8.5. The strains W03-F01^T^, S04-F03 and A2-C1_F grew between 0 and 5% (w/v) NaCl, while A2-C2_NF showed growth only up to 4% (w/v) NaCl.

**Table 3.** General properties of isolated strains W03-F01^T^ (1), S04-F03 (2), A2-C1_F (3), *B. massiliensis* DSM 110695^T^ (4) and A2-C2_NF (5), data for *B. massiliensis* taken from [14]. +, positive; −, negative

	1	2	3	4	5
Gram stain	Negative	Negative	Negative	Negative	Negative
Oxidase activity	−	−	−	−	−
Catalase activity	+	+	+	+	+
Optimum temperature (°C)	30	30	30	37	30
Temperature range (°C)	4–42	4–42	4–42	Unknown	4–45
Optimum pH	8.5	8.5	8.5	7.5*	8.5
pH range	5.5–10.5	5.5–10.5	5.5–10.5	5.0–7.5*	5.5–10.5
Maximum tolerable NaCl concentration (%)	5	5	5	<5†	4

*pH values above 7.5 not tested by [14].

†NaCl concentrations between 0 and 5 not tested by [14].

**Table 4.** Results of physiological and biochemical tests with Biolog GenIII MicroPlate Strains: W03-F01^T^ (1), S04-F03 (2), A2-C1_F (3), A2-C2_NF (4) and reference strains *B. agrestis* DSM 4586^T^ (5), *B. brennerae* DSM 9396^T^ (6), *B. ferragutiae* DSM 9390^T^ (7), *B. gaviniae* DSM 9393^T^ (8), *B. izardii* DSM 9397^T^ (9), *B. massiliensis* DSM 110695^T^ (10), *B. noackiae* DSM 9401^T^ (11), *B. warmboldiae* DSM 9404^T^ (12) and *B. selenatireducens* R73^T^ (13), data for *B. selenatireducens* R73^T^ from [13]. +, positive; −, negative; w, weak utilization/resistance; missing, no data available. Only results differing between the novel species and other members of *Buttiauxella* are shown, and complete results are given in Table S3. Results differing between strains W03-F01^T^, S04-F03, A2-C1_F and *B. massiliensis* DSM 110695^T^ are highlighted.

	1	2	3	4	5	6	7	8	9	10	11	12	13
**Carbon source utilization**													
d-Maltose	+	−	−	+	+	+	+	+	+	−	+	+	+
d-Trehalose	−	−	w	+	w	+	w	w	w	−	w	+	+
d-Turanose	w	+	w	w	+	w	w	w	w	−	w	w	
Stachyose	−	−	−	−	+	−	−	−	−	−	−	−	
d-Raffinose	−	−	−	−	+	+	w	−	+	−	−	−	
*α*-d-Lactose	w	−	−	−	w	+	w	w	+	−	−	−	+
d-Melibiose	+	w	w	−	w	+	w	−	+	−	−	−	+
*β*-Methyl-d-glucoside	−	−	−	w	w	+	w	w	+	−	−	+	+
d-Salicin	−	−	−	+	−	+	w	w	+	−	−	w	+
*N*-Acetyl-d-glucosamine	−	w	−	w	w	+	w	w	+	−	+	+	+
*N*-Acetyl-d-galactosamine	+	−	−	−	w	+	w	w	+	−	+	+	
*α*-d-Glucose	w	w	w	w	−	+	w	w	+	−	−	+	+
3-Methyl-glucose	+	+	+	+	w	−	w	+	+	−	+	+	
d-Fucose	−	−	w	−	−	−	+	−	−	−	−	−	
l-Fucose	+	−	+	−	+	−	+	−	+	−	−	+	
Inosine	w	−	w	w	+	+	w	+	+	−	+	+	+
d-Sorbitol	−	−	−	+	−	−	+	−	−	w	−	−	
d-Mannitol	−	−	−	w	w	+	w	+	+	−	w	+	+
d-Arabitol	−	−	−	−	−	+	w	+	−	−	−	−	
Myo-inositol	−	−	−	+	−	−	+	−	−	−	−	+	
d-Glucose-6-phosphate	+	+	+	+	+	+	+	+	+	+	+	−	
d-Fructose-6-phosphate	+	+	+	+	+	+	+	+	+	w	+	−	
d-Serine	+	+	+	+	−	+	+	+	−	w	+	−	
Gelatin	w	−	−	+	−	−	w	−	−	w	−	w	
l-Arginine	−	−	−	−	−	−	w	−	−	−	−	w	
l-Aspartic acid	+	+	+	+	+	+	+	+	+	−	w	+	
l-Histidine	w	−	−	−	−	−	+	−	w	−	−	w	
l-Pyroglutamic acid	−	−	−	+	−	−	+	−	w	−	−	w	
Pectin	w	−	−	−	+	−	w	+	−	−	+	+	
d-Gluconic acid	+	−	w	+	+	+	+	+	+	−	+	+	+
Mucic acid	+	+	+	−	+	+	+	+	+	−	+	−	
Quinic acid	−	w	−	−	+	+	+	+	+	−	+	+	
d-Saccharic acid	−	−	−	−	+	+	+	+	−	−	+	−	
Methylpyruvate	w	+	w	w	+	+	+	+	+	−	+	+	
d-Lactic acid methyl ester	−	−	−	−	−	−	w	w	w	−	w	−	
Citric acid	−	−	−	−	+	+	w	−	+	−	w	−	−
l-Malic acid	−	−	−	w	+	w	+	+	+	−	w	w	
Bromosuccinic acid	−	−	−	−	w	−	w	w	w	−	−	−	
Tween 40	−	−	−	w	−	−	+	+	+	−	w	+	
Acetoacetic acid	w	−	w	+	−	−	+	+	w	w	−	+	
**Chemical resistance**													
1% sodium lactate	−	w	w	w	w	w	+	w	w	−	−	−	
Fusidic acid	w	w	w	−	−	−	−	+	−	w	−	−	
d-Serine	−	w	−	−	w	w	−	+	−	−	+	−	
Troleandomycin	−	+	−	−	+	+	−	w	+	−	+	+	
Lincomycin	−	−	−	−	+	w	+	−	w	−	−	+	
Niaproof 4	+	+	+	+	+	+	+	+	+	−	+	+	
Vancomycin	+	+	+	+	+	+	+	+	+	−	+	+	
Lithium chloride	−	w	−	w	+	+	−	+	+	−	+	−	
Sodium butyrate	−	+	−	−	−	−	−	−	−	−	−	−	

**Table 5.** Fatty acid composition (%), respiratory quinones and polar lipids of strains W03-F01^T^ (1), S04-F03 (2), A2-C1_F (3) and A2-C2_NF (4) and type strains *B. agrestis* DSM 4586^T^ (5), *B. brennerae* DSM 9396^T^ (6), *B. ferragutiae* DSM 9390^T^ (7), *B. gaviniae* DSM 9393^T^ (8), *B. izardii* DSM 9397^T^ (9), *B. massiliensis* DSM 110695^T^ (10), *B. noackiae* DSM 9401^T^ (11) and *B. warmboldiae* DSM 9404^T^ (12). Major fatty acids >5% (bold: >10.0%), quinones (bold: >10%) and polar lipids (bold: main compounds) are shown. The strains were tested under the same conditions. All data were obtained from this study

Fatty acids	1	2	3	4	5	6	7	8	9	10	11	12
C_12 : 0_	3.0	3.2	3.1	1.1	3.0	3.3	1.2	3.8	2.8	3.6	3.5	4.4
C_12 : 0_ ALDE	1.4	1.2	tr	tr	1.4	1.2	1.1	1.7	1.3	1.7	tr	1.3
C_14 : 0_	6.4	7.2	6.1	7.2	5.	8.1	6.3	6.4	6.7	8.6	7.0	5.5
C_14 : 0_ 3OH	6.5	7.4	6.4	6.3	7.7	7.3	6.6	8.9	7.2	7.6	6.8	9.2
C_15 : 0_	3.3	1.2	2.8	1.6	1.7	tr	1.1	tr	34.2	tr	3.8	1.3
C_16 : 0_	36.6	37.5	35.1	38.7	35.6	39.2	38.1	30.5	7.6	40.2	36.1	33.0
C_16 : 1_ ω7c	11.6	16.0	13.9	16.4	8.8	5.8	15.5	20.0	5.8	15.2	8.6	6.2
C_17 : 0_	1.6	tr	1.4	tr	tr	tr	tr	tr	tr	–	tr	tr
C_17 : 0_ cy ω7c	19.5	17.4	19.3	19.7	23.6	24.9	21.7	13.5	26.8	13.6	24.7	27.8
C_18 : 1_ ω7c	6.6	6.1	7.5	5.1	9.3	7.1	5.8	12.3	7.0	6.4	5.6	5.4
C_19 : 0_ cy ω7c	tr	tr	tr	tr	tr	tr	tr	tr	1.9	tr	tr	3.3
Respiratory quinones	Q-8, MK‑8, Q‑7	Q-8, MK‑8, Q‑7	Q-8, MK‑8, Q‑7	Q-8, MK‑8, Q‑7	Q-8, MK‑8, Q‑7	Q-8, MK‑8, Q‑7	Q-8, MK‑8, Q‑7	Q-8, MK‑8, Q‑7	Q-8, MK‑8, Q‑7	Q-8, MK‑8, Q‑7	Q-8, MK‑8, Q‑7	Q-8, MK‑8, Q‑7
Polar lipids	DPG, PE, PG, AL, L	DPG, PE, PG, AL, L	DPG, PE, PG, AL, L	DPG, PE, PG, AL, L	DPG, PE, PG, AL, L	DPG, PE, PG, AL, L	DPG, PE, PG,	DPG, PE, PG, L	DPG, PE, PG, AL, L	DPG, PE, PG, AL, L	DPG, PE, PG, AL, L	DPG, PE, PG

–, not detected; AL, unidentified aminolipid; ALDE, aldehyde; cy, cyclopropyl; cy, cyclo; DPG, diphosphatidylglycerol; L, unidentified lipid; MK, menaquinones; PE, phosphatidylethanolamine; PG, phosphatidylglycerol; Q, ubiquinones; tr, traces.

All isolates showed respiratory activity on several substrates which were known to be used as carbon and energy sources by most *Buttiauxella* strains, e.g. dextrin, d-cellobiose, gentiobiose, d-mannose, d-fructose, d-galactose, glycerol or l-alanine (Tables 4 and S3). In contrast, they were not able to utilize, e.g. sucrose, l-histidine, *α*-ketoglutarate or propionate, which is also in line with members of the genus *Buttiauxella* [10]. Strains W03-F01^T^, S04-F03 and A2-C1_F showed no respiratory activity on *β*-methyl-d-glucoside, d-salicin, d-saccharic acid and l-malic acid which were used by most of the known *Buttiauxella* strains so far [10].

Intra-species variation between strains W03-F01^T^, S04-F03 and A2-C1_F was observed for the utilization of *N*-acetyl-d-galactosamine, l-fucose, d-gluconic acid and d-maltose.

Compared to strains W03-F01^T^, S04-F03 and A2-C1_F, *B. massiliensis* DSM 110695^T^ does not utilize d-turanose, d-melibiose, 3-methyl-glucose, l-aspartic acid, mucic acid and methylpyruvate, in addition to the absence of *β*-d-glucuronidase activity.

The strains W03-F01^T^, S04-F03 and A2-C1_F were found to be resistant towards fusidic acid, rifamycin SV, guanidine HCl, Niaproof 4, vancomycin, tetrazolium blue and violet and aztreonam but were susceptible against minocycline, lincomycin, nalidixic acid and sodium bromate (Tables 4 and S3). Specifically, resistance against Niaproof 4 and vancomycin is in line with other members of the genus *Buttiauxella* but differentiates strains W03-F01^T^, S04-F03 and A2-C1_F from *B. massiliensis* DSM 110695^T^.

As shown in Table 5, strains W03-F01^T^, S04-F03 and A2-C1_F contained C_16 : 0_, C_16 : 1_ ω7c and C_17 : 0_ cyclo ω7c as the major fatty acids, alongside smaller amounts of C_14 : 0_, C_14 : 0_ 3OH and C_18 : 1_ ω7c. The fatty acid compositions are consistent with the fatty acid profiles known for the genus *Buttiauxella* [51]. Ubiquinone Q8 was the predominant respiratory quinone of all strains, accompanied with minor amounts of Q7 and menaquinone MK8. Diphosphatidylglycerol, phosphatidylethanolamine and phosphatidylglycerol were the predominant polar lipids. Additionally, all strains, except *B. ferragutiae* DSM 9390^T^, and *B. warmboldiae* DSM 9404^T^ also contained minor amounts of unidentified lipids and/or aminolipids. Overall, the chemotaxonomic markers resemble those known for members of the genus *Buttiauxella* [10].

## Taxonomic conclusion

Based on the polyphasic approach, the three strains W03-F01^T^, S04-F03 and A2-C1_F represent a novel species of the genus *Buttiauxella* for which we propose the name *B. cochleicola* sp. nov. The strains have been isolated from environmental (non-human) sources such as drinking water (A2-C1_F) and faeces of gastropods (W03-F01^T^, S04-F03). The new species is of environmental relevance, as isolates have been obtained from faecal samples of several snails and slugs, like *Arion vulgaris*, *Helix pomatia*, *Cepaea hortensis* and *Cepaea nemoralis*, and the species is likely to be present in other poikilothermic animals as well [6, 52].

The new species is unusual, as it possesses the enzyme *β*-d-glucuronidase (encoded by *uidA*), an enzyme considered indicative for *E. coli*. Thus, these *Buttiauxella* isolates can lead to false-positive *E. coli* results in drinking water analysis [6]; therefore, designation of the new species is also of relevance for the practice of drinking water analysis. *β*-d-Glucuronidase activity has also been demonstrated in two other *Buttiauxella* strains, namely *B. noackiae* MCE (originally published as *B. agrestis*) and *B. noackiae* 11 A-P3, isolated from surface water and oysters, respectively [3], yet it is absent in all other *Buttiauxella* strains, including the type strain *B. noackiae* DSM 9401^T^ and the closer related strains *B. gaviniae* DSM 9393^T^ and *B. massiliensis* DSM 110695^T^.

The closest relative is *B. massiliensis* DSM 110695^T^, yet, in contrast to the hereby described species, *B. massiliensis* has been isolated from a medical sample (open human bone fracture). In addition to the absence of *β*-d-glucuronidase activity, *B. massiliensis* does not utilize d-turanose, d-melibiose, 3-methyl-glucose, l-aspartic acid, mucic acid and methylpyruvate, thus, showing considerable differences in biochemical testing. Furthermore, *B. massiliensis* DSM 110695^T^ has an optimal growth temperature of 37 °C (vs. 30 °C for the strains W03-F01^T^, S04-F03 and A2-C1_F) [14]. In combination, this polyphasic approach leads us to propose that *B. massiliensis* DSM 110695^T^ and the three newly isolated strains W03-F01^T^, S04-F03 and A2-C1_F represent different and distinguishable species.

## Description of *Buttiauxella cochleicola* sp. nov.

*Buttiauxella cochleicola* sp. nov. (coch.le.i’co.la. L. fem. n. *cochlea*, snail; L. masc./fem. suff. -*cola*, inhabitant, dweller; from L. masc./fem. n. *incola*, dweller; N.L. fem. n. *cochleicola*, inhabitant of the snail).

Cells are Gram-negative, non-spore-forming rods, ~1 µm wide and 2–4 µm long, depending on growth stage. They are facultative anaerobic, oxidase-negative and catalase-positive. They exhibit *β*-d-glucuronidase activity. Good growth occurs on different nutrient agars, like HPC agar, brain heart infusion agar, tryptic soy agar and Reasoner’s 2A agar or on selective agars for coliform bacteria, like chromogenic coliform agar. Growth occurs between 4 and 42 °C in the presence of 0 up to 5% (w/v) NaCl. Optimal growth temperature is 30 °C, and the optimal pH is 8.5.

Strains W03-F01^T^, S04-F03 and A2-C1_F show respiratory activity on the following substrates: acetic acid, *N*-acetyl-*β*-d-mannosamine, *N*-acetyl-neuraminic acid, l-alanine, l-aspartate, d-cellobiose, dextrin, d-fructose-6-phosphate, d-fructose, l-galactonic acid lactone, d-galactose, d-galacturonic acid, gentiobiose, *α*-d-glucose, glucuronamide, d-glucuronic acid, d-glucose-6-phosphate, l-glutamate, glycerol, glycyl-l-proline, lactic acid, d-mannose, d-melibiose, 3-methylglucose, methylpyruvate, mucic acid, l-rhamnose, d-serine, l-serine and d-turanose.

No utilization is observed for *γ*-aminobutyric acid, d-arabitol, l-arginine, d-aspartate, bromosuccinate, citrate, formic acid, *α*-hydroxybutyric acid, *β*-hydroxy-d,l-butyric acid, *p-*hydroxyphenylacetate, *α*-ketobutyric acid, *α*-ketoglutaric acid, d-lactic acid methylester, d-malate, l-malate, d-mannitol, *β*-methyl-d-glucoside, myo-inositol, l-pyroglutamic acid, d-raffinose, d-saccharic acid, d-salicin, stachyose, d-sorbitol, sucrose and Tween 40.

The major fatty acids (>10%) are C_16 : 0_, C_16 : 1_ ω7c and C_17 : 0_ cyclo ω7c. The major respiratory quinone is Q-8, and minor compounds are MK-8 and Q-7. The polar lipid profile mainly consists of DPG, PG and PE, among unidentified aminolipids and unidentified lipids.

The type strain is W03-F01^T^ (=DSM 113920^T^=CIP 112567^T^), which was isolated from a faecal sample from the snail *Helix pomatia* of a small village in south-western Germany. Strains S04-F03 (=DSM 113932=CIP 112568) and A2-C1_F (=DSM 113933=CIP 112569) represent further strains of the species isolated from a faecal sample of the snail *Arion vulgaris* and from a drinking water sample, respectively. The 16S rRNA gene sequence is PV742342. The genome of the type strain (JAJSOT000000000) is characterized by a size of 4.79 Mbp and a G+C content of 50.0 mol%.

## Supplementary material

10.1099/ijsem.0.007265 Supplementary Material 1.

10.1099/ijsem.0.007265 Supplementary Material 2.

## Abbreviations

ANI: average nucleotide identity
dDDH: digital DNA–DNA hybridization
FAMEs: fatty acid methyl esters
GBDP: Genome blast Distance Phylogeny
HPC: heterotrophic plate count
