# Supplementary material for: Buttiauxella cochleicola sp. nov., a novel coliform bacterium with β-d-glucuronidase activity, isolated from gastropods and drinking water
Source: Int J Syst Evol Microbiol. 2026 Aug 13;76(8):007265. doi: 10.1099/ijsem.0.007265 (PMC13472201; doi:10.1099/ijsem.0.007265)
Supplement: Supplementary file 1 [file ijsem-76-07265-s001.pdf]

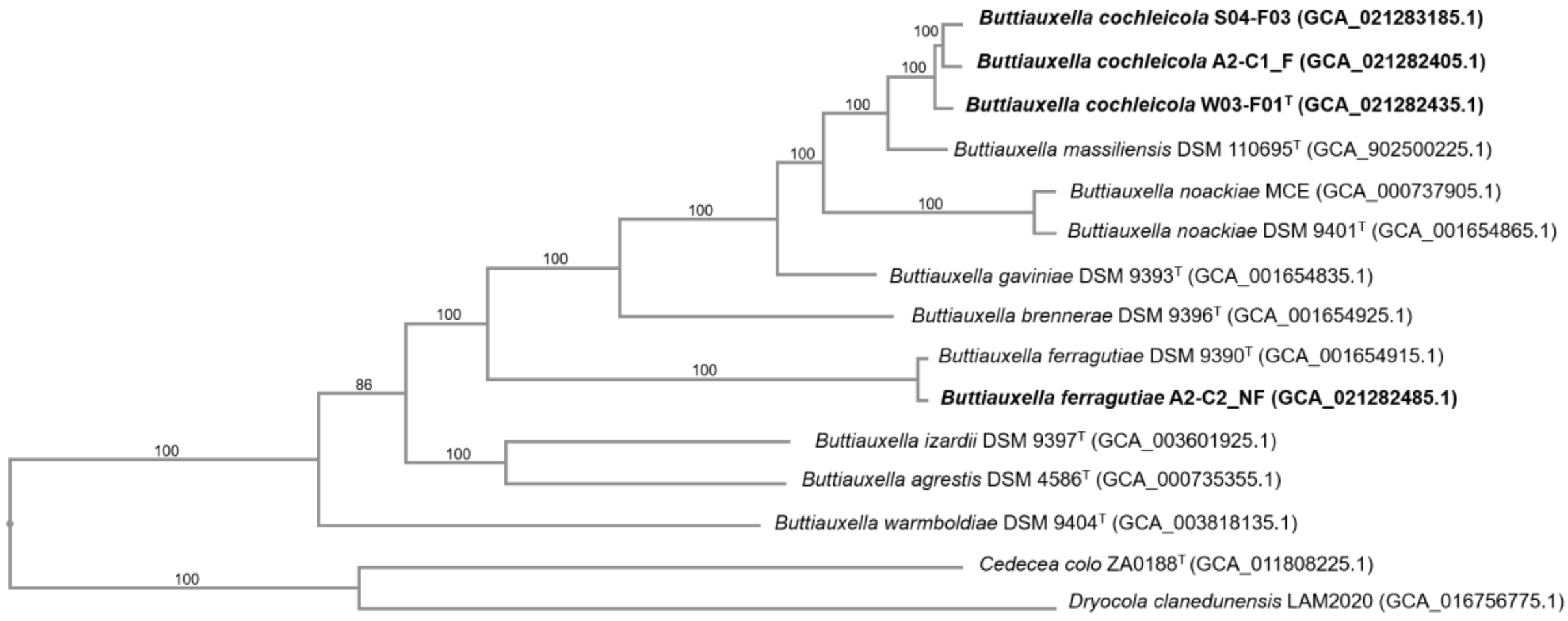

Fig. S1. Genome-based phylogeny calculated with the codon-tree pipeline in BV-BRC. The tree from 1000 genes was generated using single-copy cross-genera protein families analyzing aligned proteins and coding DNA from single-copy genes with RAxML.

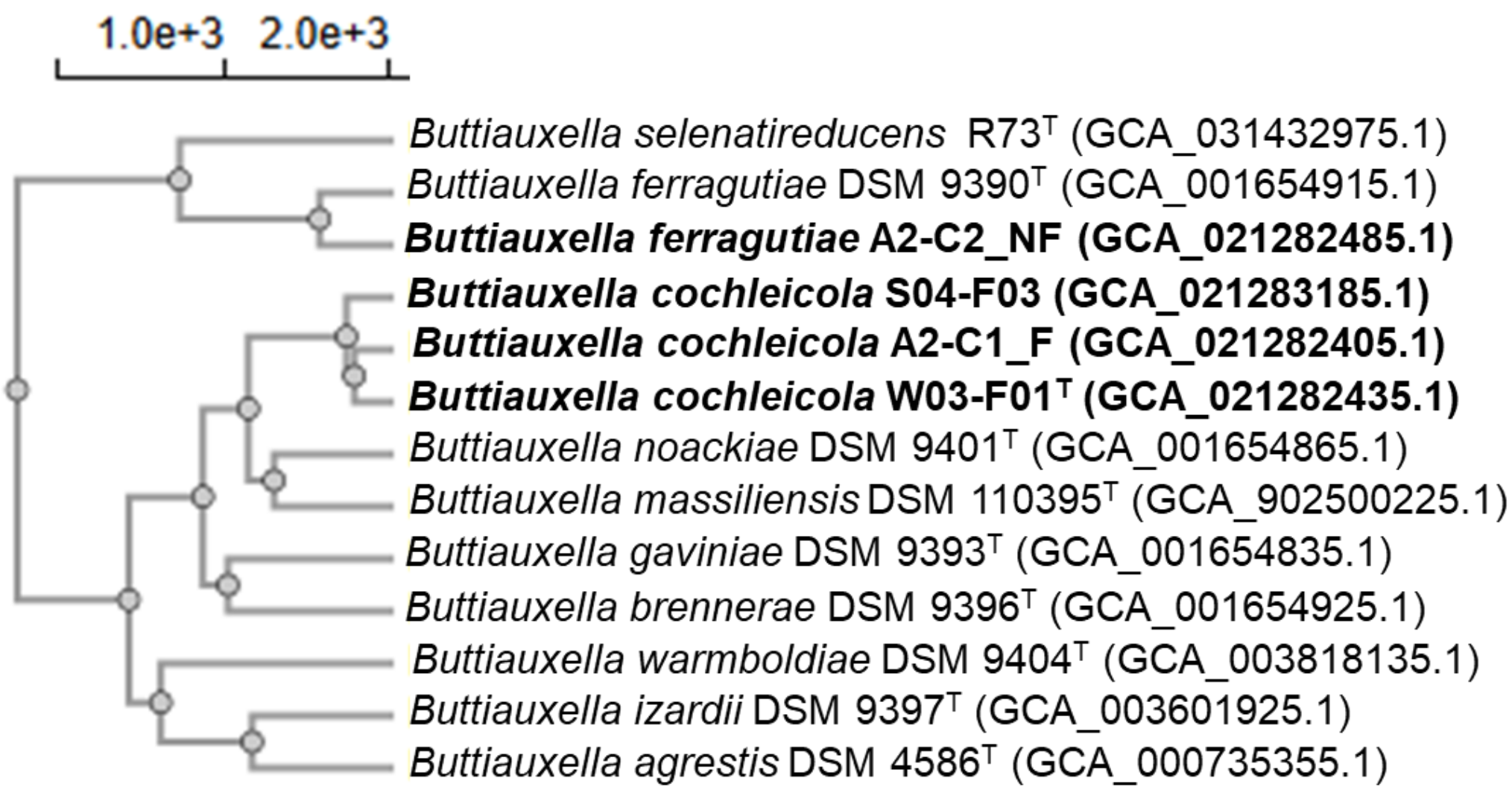

Fig. S2. Pan-genome-based phylogeny calculated with the PanExplorer application (Dereeper et al. 2022).

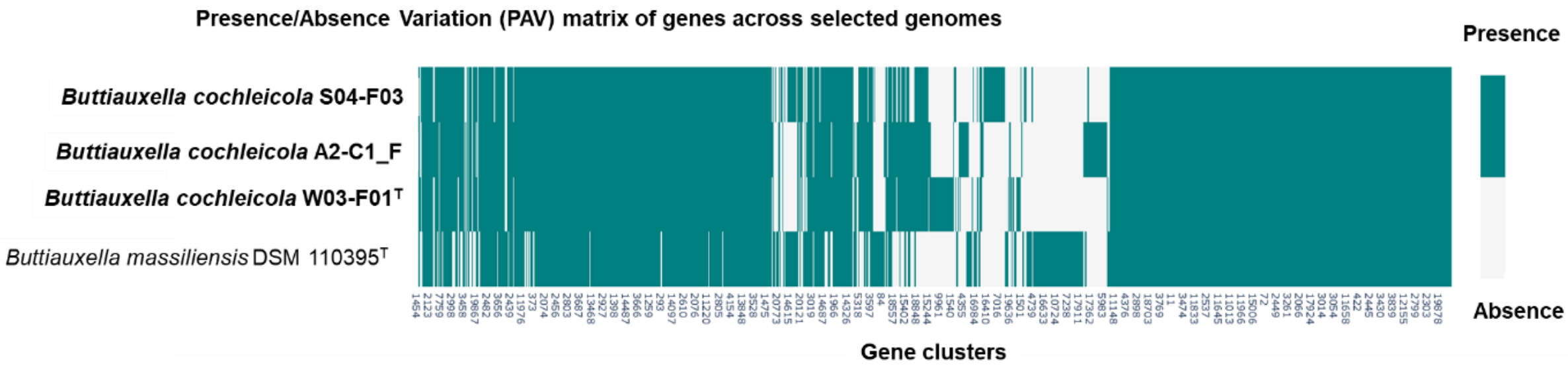

Fig. S3. Pan-genome visualization of gene presence/absence of genomes *B. cochleicola* strains W03-F01^T, S04-F03, A2-C1_F and *B. massiliensis* DSM 110695^T (Dereeper et al. 2022). 325 genes were detected in *B. cochleicola* and absent in *B. massiliensis*, whereas 553 genes were only present in *B. massiliensis*.

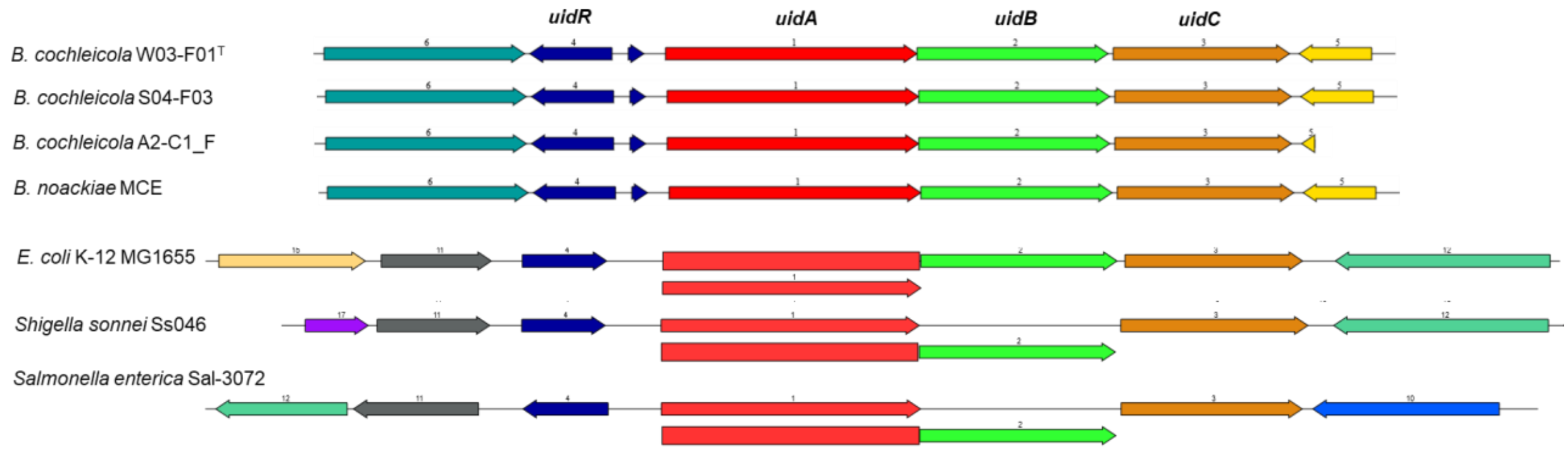

Fig. S4. Genomic context of *uidA* gene in *Buttiauxella cochleicola* strains W03-F01^{T}, S04-F03 and A2-C1_F, *Buttiauxella noackiae* strain MCE, *Escherichia coli* strain K-12 MG1655, *Shigella sonnei* strain Ss046 and *Salmonella enterica* strain Sal-3072. *uidR*: transcriptional repressor, *uidA*: β-D-glucuronidase, *uidB*: glucuronide transporter, *uidC*: glucuronide transport facilitator.

Table S1. Completeness and contamination of the draft genomes.

Bacterial species	Strain	Completeness	Contamination
Buttiauxella cochleicola	W03-F01^T	100%	0.72%
Buttiauxella cochleicola	S04-F03	100%	0.72%
Buttiauxella cochleicola	A2-C1_F	100%	1.13%
Buttiauxella ferragutiae	A2-C2_NF	100%	0.39%

Table S2. Pan-genes table (Presence-absence Variation, PAV Matrix → see additional .xlsx file)

Table S3. Results of physiological and biochemical tests with Biolog GenIII MicroPlate. Strains: W03-F01^T (1), S04-F03 (2), A2-C1_F (3), A2-C2_NF (4) and reference strains *B. agrestis* DSM 4586^T (5), *B. brennerae* DSM 9396^T (6), *B. ferragutiae* DSM 9390^T (7), *B. gaviniae* DSM 9393^T (8), *B. izardii* DSM 9397^T (9), *B. massiliensis* DSM 110695^T (10), *B. noackiae* DSM 9401^T (11), *B. warmboldiae* DSM 9404^T (12), and *B. selenatireducens* R73^T (13), data for *B. selenatireducens* R73^T from Zhang et al. 2024. +: positive, -: negative, w: weak utilization/resistance, missing: no data available.

	**1**	**2**	**3**	**4**	**5**	**6**	**7**	**8**	**9**	**10**	**11**	**12**	**13**
**Carbon source utilization**													
Dextrin	+	+	+	+	+	+	+	+	+	+	+	+	
D-Maltose	+	-	-	+	+	+	+	+	+	-	+	+	+
D-Trehalose	-	-	w	+	w	+	w	w	w	-	w	+	+
D-Cellobiose	+	+	+	+	+	+	+	+	+	w	+	+	+
Gentiobiose	+	+	+	+	+	+	+	+	+	+	+	+	+
Sucrose	-	-	-	-	-	-	-	-	-	-	-	-	-
D-Turanose	w	+	w	w	+	w	w	w	w	-	w	w	
Stachyose	-	-	-	-	+	-	-	-	-	-	-	-	
D-Raffinose	-	-	-	-	+	+	w	-	+	-	-	-	
α-D-Lactose	w	-	-	-	w	+	w	w	+	-	-	-	+
D-Melibiose	+	w	w	-	w	+	w	-	+	-	-	-	+
β-Methyl-D-glucoside	-	-	-	w	w	+	w	w	+	-	-	+	+
D-Salicin	-	-	-	+	-	+	w	w	+	-	-	w	+
N-Acetyl-D-glucosamine	-	w	-	w	w	+	w	w	+	-	+	+	+
N-Acetyl-β-D-mannosamine	+	+	+	+	+	+	+	+	+	+	+	+	
N-Acetyl-D-galactosamine	+	-	-	-	w	+	w	w	+	-	+	+	
N-Acetyl-neuraminic acid	+	+	+	+	+	+	+	+	+	+	+	+	
α-D-Glucose	w	w	w	w	-	+	w	w	+	-	-	+	+
D-Mannose	w	+	+	+	w	+	w	+	+	w	+	+	+
D-Fructose	w	+	w	+	w	+	+	+	+	w	+	+	+
D-Galactose	+	+	+	+	w	+	w	+	+	w	+	+	+
3-Methyl-glucose	+	+	+	+	w	-	w	+	+	-	+	+	
D-Fucose	-	-	w	-	-	-	+	-	-	-	-	-	
L-Fucose	+	-	+	-	+	-	+	-	+	-	-	+	
L-Rhamnose	+	+	+	+	w	+	w	+	+	w	+	+	+
Inosine	w	-	w	w	+	+	w	+	+	-	+	+	+
D-Sorbitol	-	-	-	+	-	-	+	-	-	w	-	-	
D-Mannitol	-	-	-	w	w	+	w	+	+	-	w	+	+
D-Arabitol	-	-	-	-	-	+	w	+	-	-	-	-	
Myo-inositol	-	-	-	+	-	-	+	-	-	-	-	+	
Glycerol	+	+	+	+	+	+	+	+	+	+	+	+	+

D-Glucose-6-phosphate	+	+	+	+	+	+	+	+	+	+	+	-	
D-Fructose-6-phosphate	+	+	+	+	+	+	+	+	+	w	+	-	
D-Aspartic acid	-	-	-	-	-	-	-	-	-	-	-	-	
D-Serine	+	+	+	+	-	+	+	+	-	w	+	-	
Gelatin	w	-	-	+	-	-	w	-	-	w	-	w	
Glycyl-L-proline	+	+	+	+	+	+	+	+	+	+	+	+	
L-Alanine	+	+	+	+	+	+	+	+	+	+	+	+	
L-Arginine	-	-	-	-	-	-	w	-	-	-	-	w	
L-Aspartic acid	+	+	+	+	+	+	+	+	+	-	w	+	
L-Glutamic acid	+	+	+	+	+	+	+	+	+	+	w	+	
L-Histidine	w	-	-	-	-	-	+	-	w	-	-	w	
L-Pyroglutamic acid	-	-	-	+	-	-	+	-	w	-	-	w	
L-Serine	+	+	+	+	+	+	+	+	+	+	+	+	
Pectin	w	-	-	-	+	-	w	+	-	-	+	+	
D-Galacturonic acid	+	+	+	+	+	+	+	+	+	+	+	+	
L-Galactonic acid lactone	+	+	+	+	+	+	+	+	+	+	+	+	
D-Gluconic acid	+	-	w	+	+	+	+	+	+	-	+	+	+
D-Glucuronic acid	+	+	+	+	+	+	+	+	+	w	+	+	
Glucuronamide	+	+	+	+	+	+	+	+	+	w	+	+	
Mucic acid	+	+	+	-	+	+	+	+	+	-	+	-	
Quinic acid	-	w	-	-	+	+	+	+	+	-	+	+	
D-Saccharic acid	-	-	-	-	+	+	+	+	-	-	+	-	
p-Hydroxy-phenylacetic acid	-	-	-	-	-	-	w	-	-	-	-	-	
Methylpyruvate	w	+	w	w	+	+	+	+	+	-	+	+	
D-Lactic acid methylester	-	-	-	-	-	-	w	w	w	-	w	-	
L-Lactic acid	+	+	+	+	+	+	+	+	+	w	+	+	
Citric acid	-	-	-	-	+	+	w	-	+	-	w	-	-
α-Ketoglutaric acid	-	-	-	-	-	-	w	-	-	-	-	-	
D-Malic acid	-	-	-	-	-	-	-	w	-	-	-	-	
L-Malic acid	-	-	-	w	+	w	+	+	+	-	w	w	
Bromosuccinic acid	-	-	-	-	w	-	w	w	w	-	-	-	
Tween 40	-	-	-	w	-	-	+	+	+	-	w	+	
γ-Aminobutyric acid	-	-	-	-	-	-	-	-	-	-	-	-	
α-Hydroxybutyric acid	-	-	-	-	-	-	-	-	-	-	-	-	
β-Hydroxy-D,L-butyric acid	-	-	-	-	-	-	-	-	-	-	-	-	
α-Ketobutyric acid	-	-	-	-	-	-	-	-	-	-	-	-	
Acetoacetic acid	w	-	w	+	-	-	+	+	w	w	-	+	
Propionic acid	-	w	-	-	-	-	-	-	-	-	-	-	
Acetic acid	+	+	+	+	+	+	+	+	+	+	+	+	
Formic acid	-	-	-	-	-	-	-	-	-	-	-	-	

Table S3 continued.

	1	2	3	4	5	6	7	8	9	10	11	12	13
**Chemical resistance**													
pH 6	+	+	+	+	+	+	+	+	+	+	+	+	
pH 5	-	w	w	w	w	-	-	-	-	-	-	-	
1 % Sodium lactate	-	w	w	w	w	w	+	w	w	-	-	-	
Fusidic acid	w	w	w	-	-	-	-	+	-	w	-	-	
D-Serine	-	w	-	-	w	w	-	+	-	-	+	-	
Troleandomycin	-	+	-	-	+	+	-	w	+	-	+	+	
Rifamycin SV	+	+	+	+	+	+	+	+	+	w	+	+	
Minocycline	-	-	-	-	-	-	-	-	-	-	-	-	
Lincomycin	-	-	-	-	+	w	+	-	w	-	-	+	
Guanidine HCl	+	+	+	+	+	+	+	+	+	w	+	+	
Niaproof 4	+	+	+	+	+	+	+	+	+	-	+	+	
Vancomycin	+	+	+	+	+	+	+	+	+	-	+	+	
Tetrazolium violet	+	+	+	+	+	+	+	+	+	+	+	+	
Tetrazolium blue	+	+	+	+	+	+	+	+	+	+	+	+	
Nalidixic acid	-	-	-	-	w	-	-	-	-	-	-	-	
Lithium chloride	-	w	-	w	+	+	-	+	+	-	+	-	
Potassium tellurite	-	-	-	w	-	-	-	-	-	-	-	-	
Aztreonam	+	+	+	+	+	+	+	+	+	w	+	+	
Sodium butyrate	-	+	-	-	-	-	-	-	-	-	-	-	
Sodium bromate	-	-	-	-	-	-	-	-	-	-	-	-	
